# Perfect duet: Dual recombinases improve genetic resolution

**DOI:** 10.1111/cpr.13446

**Published:** 2023-04-14

**Authors:** Hongxin Li, Wendong Weng, Bin Zhou

**Affiliations:** ^1^ State Key Laboratory of Cell Biology, Shanghai Institute of Biochemistry and Cell Biology, Center for Excellence in Molecular Cell Science, Chinese Academy of Sciences University of Chinese Academy of Sciences Shanghai China; ^2^ Key Laboratory of Systems Health Science of Zhejiang Province School of Life Science, Hangzhou Institute for Advanced Study, University of Chinese Academy of Sciences Hangzhou China; ^3^ School of Life Science and Technology ShanghaiTech University Shanghai China; ^4^ New Cornerstone Science Laboratory Shenzhen China

## Abstract

As a powerful genetic tool, site‐specific recombinases (SSRs) have been widely used in genomic manipulation to elucidate cell fate plasticity in vivo, advancing research in stem cell and regeneration medicine. However, the low resolution of conventional single‐recombinase‐mediated lineage tracing strategies, which rely heavily on the specificity of one marker gene, has led to controversial conclusions in many scientific questions. Therefore, different SSRs systems are combined to improve the accuracy of lineage tracing. Here we review the recent advances in dual‐recombinase‐mediated genetic approaches, including the development of novel genetic recombination technologies and their applications in cell differentiation, proliferation, and genetic manipulation. In comparison with the single‐recombinase system, we also discuss the advantages of dual‐genetic strategies in solving scientific issues as well as their technical limitations.

## Graphical abstract

To improve the resolution of lineage tracing and genetic manipulation, the dual‐recombinase system works in a variety of ways. The OR‐, AND‐, and NOT‐logic‐based strategies identify a specific cell population more precisely compared to the single‐recombinase‐mediated approaches. In addition, seamless recording of gene activation and cell fate transitions can also be achieved in dual‐genetic system.

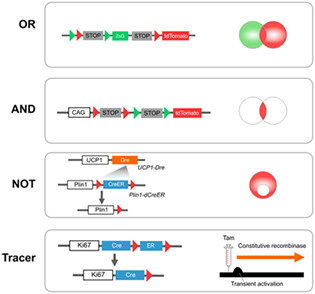

## 1 INTRODUCTION

Lineage tracing is the identification of all descendants of the marked founder cells, which provides information about the progeny status.^1^ Typically, by performing immunofluorescence staining or in situ hybridization, the spatial distribution, proliferation and differentiation properties of offspring cells are presented. As a powerful tool in stem cell research, lineage tracing promotes our understanding of progenitor cell fate determination in embryonic development, tissue maintenance of homeostasis, tissue repair and regeneration in disease.^2^, ^3^ The crucial point, however, is how to accurately target the interested cells without unintended labelling, which is largely dependent on the capacity of genetic tools mediated by site‐specific recombinases (SSRs).

Cre‐*loxP* is one of the most widely used recombinase systems in lineage tracing. Cre recombinase is a 38KD protein originated from P1 bacteriophage, catalysing the recombination between two 34 bp sequences, *loxP*.^4^ In the system, Cre targets the *loxP* sites specifically and excises the flanked elements (e.g., *stop* cassette) between two *loxP* in the same direction, unlocking the expression of the reporter behind the *loxP*; similarly, Dre‐*rox*,^5^ Flp‐*frt*,^6^ Nigri‐*nox*,^7^ VCre‐*VloxP* and so on.^8^ Due to the non‐reversible and heritable recombination, all descendant cells express the reporter gene no matter what kind of subsequent cell fate transition events, transdifferentiation or proliferation.^9^ In addition, in order to achieve temporal control, the ligand‐binding domain of human oestrogen receptor (ER) is fused to Cre.^10^ The compound CreER combines the heat shock protein 90 (HSP90) to keep inactivate in the cytoplasm. After Tamoxifen (Tam) treatment, CreER dissociates from HSP90 and translocates into the nucleus to work on the *loxP* sites. Thus, the inducible CreER can be extensively used to explore cell fate in a specific time window.

With a reporter line, Cre expression controlled by a cell‐specific promoter allows selective labelling of the cell population of interest. In this scenario, one tends to ideally consider that the reporter is exclusively expressed in a specific cell type. However, unanticipated activation of the reporter in other cell lineages, referred to as ‘ectopic’ expression, usually produces contradictory conclusions.^11^, ^12^ Actually, it is hard for a marker gene to be highly specific to one cell population. On the other hand, a single marker is unable to identify one cell type and its sub‐populations.^12^, ^13^ As a consequence, additional iterations of conventional single‐recombinase methods are considerably needed to realize more precise lineage tracing.^14^ By combining distinct and orthogonal recombinases, dual‐recombinase‐mediated genetic approaches have been developed to precisely label cell subtypes, capture transient gene activation, and perform genetic manipulation, among others. In this review, we discuss the working principles of dual‐recombinase‐mediated genetic approaches in exploring cell fate plasticity, and how to orchestrate different recombinases to resolve controversial issues with higher resolution than the single‐recombinase system.

## 2 DUAL‐RECOMBINASE‐MEDIATED LINEAGE TRACING VIA OR‐LOGIC

The conventional lineage‐tracing tool greatly relies on the specificity of the marker gene, but the expression pattern of the gene might not be well documented, take Cre‐*loxP* for example, there may be stochastic and unexpected Cre activation in the germline or during early development.^15^ Furthermore, possibly the assumed ‘specific’ promoter is not specific because a single marker is not sufficient to define one cell type.^13^, ^16^ Nowadays, more precise expression maps of genes can be illuminated with the help of single‐cell RNA sequencing (scRNA‐seq). Meanwhile, new cell markers are expected to be identified. But the key point is how imperfectly ‘specific’ cell markers can be utilized to precisely trace cell fate in certain scientific issues. For unbiased lineage tracing, researchers introduced Dre‐*rox* into the Cre‐*loxP* system.^14^ The OR‐logic implies targeting the cell subpopulation negative for gene *A* of the other subpopulation when both subpopulations express the common gene *A*, which is especially helpful when there are no known markers for a subpopulation. Here we categorize the applications of the dual‐recombinase system in solving some controversial questions by more precise fate maps.

### 2.1 Precise fate‐mapping of cardiac myocytes and nonmyocytes

**FIGURE 1:**
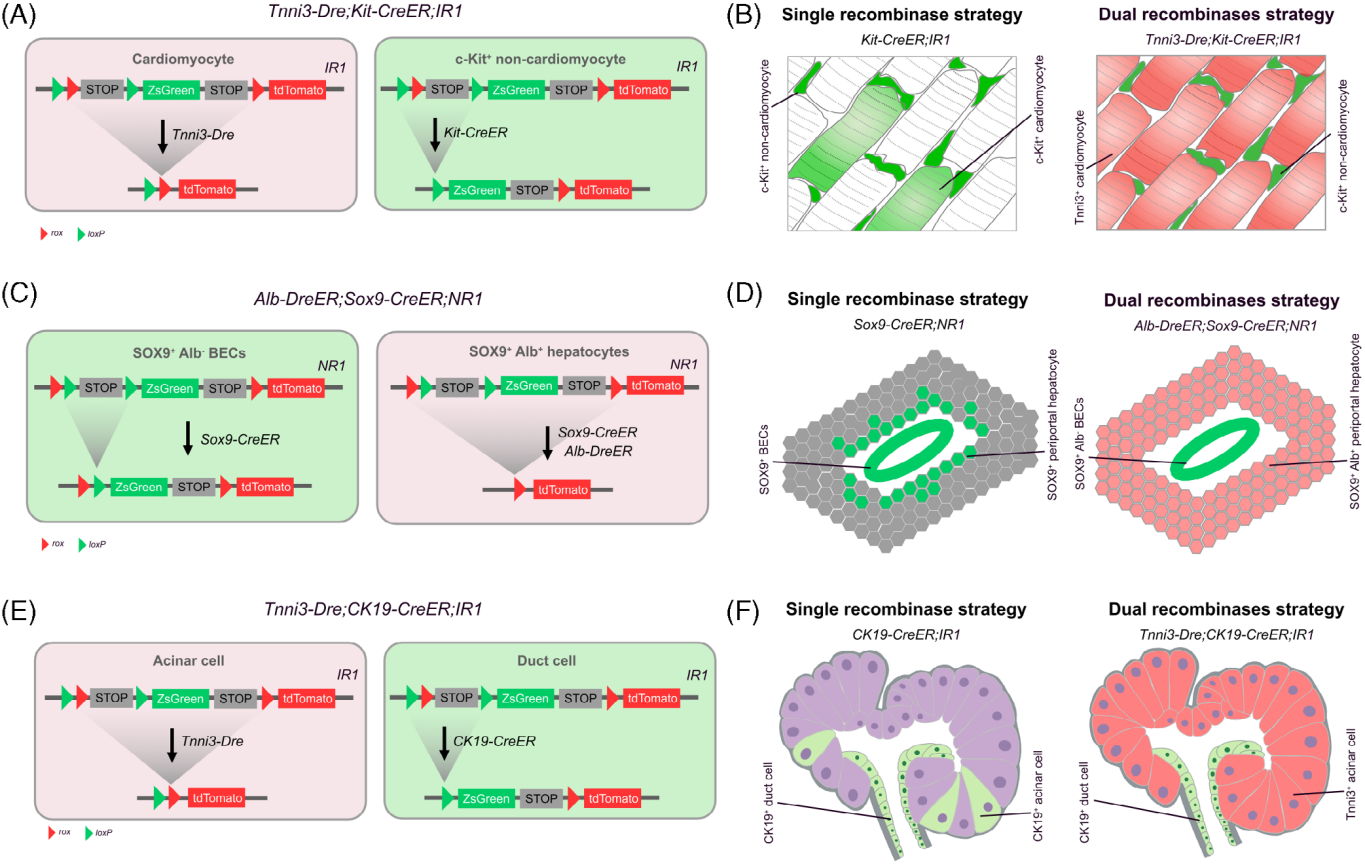
Mechanisms and examples of OR‐logic actualization. (A,B) *Kit‐CreER* is active in both non‐CMs and CMs. To block *Kit‐CreER* labelling in CMs, constitutive *Tnni3‐Dre* induces Dre‐*rox* recombination and removes the *loxP* site for Cre‐recombination. (C,D) The working principle of the nested reporter (*NR1*) is that the inducible Dre‐mediated recombination removes the Cre‐induced ZsGreen expression, which allows the precise labelling of BECs without targeting any hepatocytes. (E,F) *IR1* is used to precisely label pancreatic ductal cells without contaminating acinar cells.

The existence of endogenous cardiac stem cells (CSCs) in the adult heart is fascinating to cardiomyocyte regeneration after injury. However, imprecise genetic tools have made the existence of CSCs contentious. c‐Kit^+^ cells were reported as CSCs and contributed to myocyte renewal after cardiac damage,^17^ whereas other studies argued that c‐Kit^+^ cells harbour minimally myogenic potential of differentiation.^18^, ^19^, ^20^, ^21^ Subsequent studies clarified that it is the unintended labelling of c‐Kit^+^ cardiomyocytes that leads to the misinterpretation of *Kit‐CreER* lineage.^18^ To overcome the technical hurdle of non‐specific recombination, an interleaved reporter line was established, named *DeaLT‐IR* (dual‐recombinase‐activated lineage tracing with interleaved reporter) (Figure 1B).^14^ Combined with *IR1*, *Tnni3‐Dre* first and specifically secures the Dre‐*rox* recombination in cardiomyocytes, simultaneously precluding Cre‐*loxP*‐mediated unwanted labelling of cardiomyocytes. And *Kit‐CreER*‐mediated Cre‐*loxP* recombination leads to ZsGreen expression in c‐Kit^+^ non‐cardiomyocytes after Tam induction (Figure 1A). Based on the OR‐logic‐mediated strategy, no ZsGreen^+^ cardiomyocytes were detected after cardiac injury, disproving the CSC potential of *c‐Kit* non‐cardiomyocytes in mammalian adult hearts. In addition to *c‐Kit*, there could be other putative CSCs markers,^22^, ^23^, ^24^, ^25^ even some unknown and undefined. Another research used three different *IR* lines and the nested reporter *DeaLT‐NR* to distinctly label cardiomyocytes and non‐myocytes simultaneously, and four lineage tracing strategies independently revealed that there is no nonmyocyte‐to‐myocyte conversion in the adult heart.^26^

### 2.2 Tracing the Sox9^+^ biliary epithelial cell fate transition

SOX9 has been reported to be expressed in biliary epithelial cells as well as a subset of periportal hepatocytes.^27^ Hence, to reveal whether SOX9^+^ BECs transdifferentiate into hepatocytes in injury, new genetic tools should be developed to unambiguously label BECs and hepatocytes. The aforementioned *DeaLT‐IR1* permits sequential recombination, whereas *DeaLT‐NR* allows simultaneous expression of *CreER* and *DreER*, still the Dre‐*rox* system rigorously controls Cre‐*loxP* recombination.^14^ According to the OR‐logic, *Alb‐DreER;Sox9‐CreER;NR1* mice were generated, in which Cre was activated in both Sox9^+^ BECs and Sox9^+^ periportal hepatocytes upon Tam injection, while Dre‐*rox* recombination only occurs in Alb^+^ periportal hepatocytes.^14^ As a result, SOX9^+^Alb^−^ BECs were tagged as ZsGreen^+^, while under *Alb* activation, Dre removes ZsGreen and the *stop* cassette, unlocking the expression of tdTomato specifically in SOX9^+^Alb^+^ hepatocytes (Figure 1C,D). In vivo genetic evidence indicated that SOX9^+^ BECs do not give rise to de novo hepatocytes in homeostasis or injury.

### 2.3 Lineage transdifferentiation between acinar cells and ductal cells

In the research of cell fate conversion among exocrine cells, previous studies have been contradictory about the contribution of ductal cells to acinar cells in adults,^28^, ^29^, ^30^ as is the case with acinar‐to‐ductal metaplasia.^31^, ^32^ Based on a sequential recombination system, *Tnni3‐Dre;CK19‐CreER;IR1* mice were generated to label ductal cells and acinar cells with different fluorescent proteins.^33^ Given that CK19 is expressed in the majority of ductal cells and few acinar cells, constitutive Dre‐mediated Dre‐*rox* recombination preferentially and specifically occurs in Tnni3^+^ acinar cells, preventing the ‘ectopic’ labelling by *CK19‐CreER* in acinar cells. In this way, the two types of cells can be labelled with distinct genetic markers simultaneously (Figure 1E, F). After that, acinar‐to‐ductal conversion was proved in the pathologic models of pancreatic ductal ligation and pancreatitis. The ductal‐to‐acinar transition has also been found upon extreme acinar cell loss.

## 3 ORTHOGONAL RECOMBINASES DECIPHER CELL FATE VIA AND‐LOGIC

A specific cell population tends to be defined by more than one maker sometimes.^13^ AND‐logic strategies, which take the intersection of different cell lineages represented by different markers, have been devised to accurately decipher cell fate in homeostasis and pathologic state. Here we categorize the applications in tracing cell populations in different scenes using the orthogonal recombinase systems.

### 3.1 Tracing bronchioalveolar stem cells in lung regeneration

**FIGURE 2:**
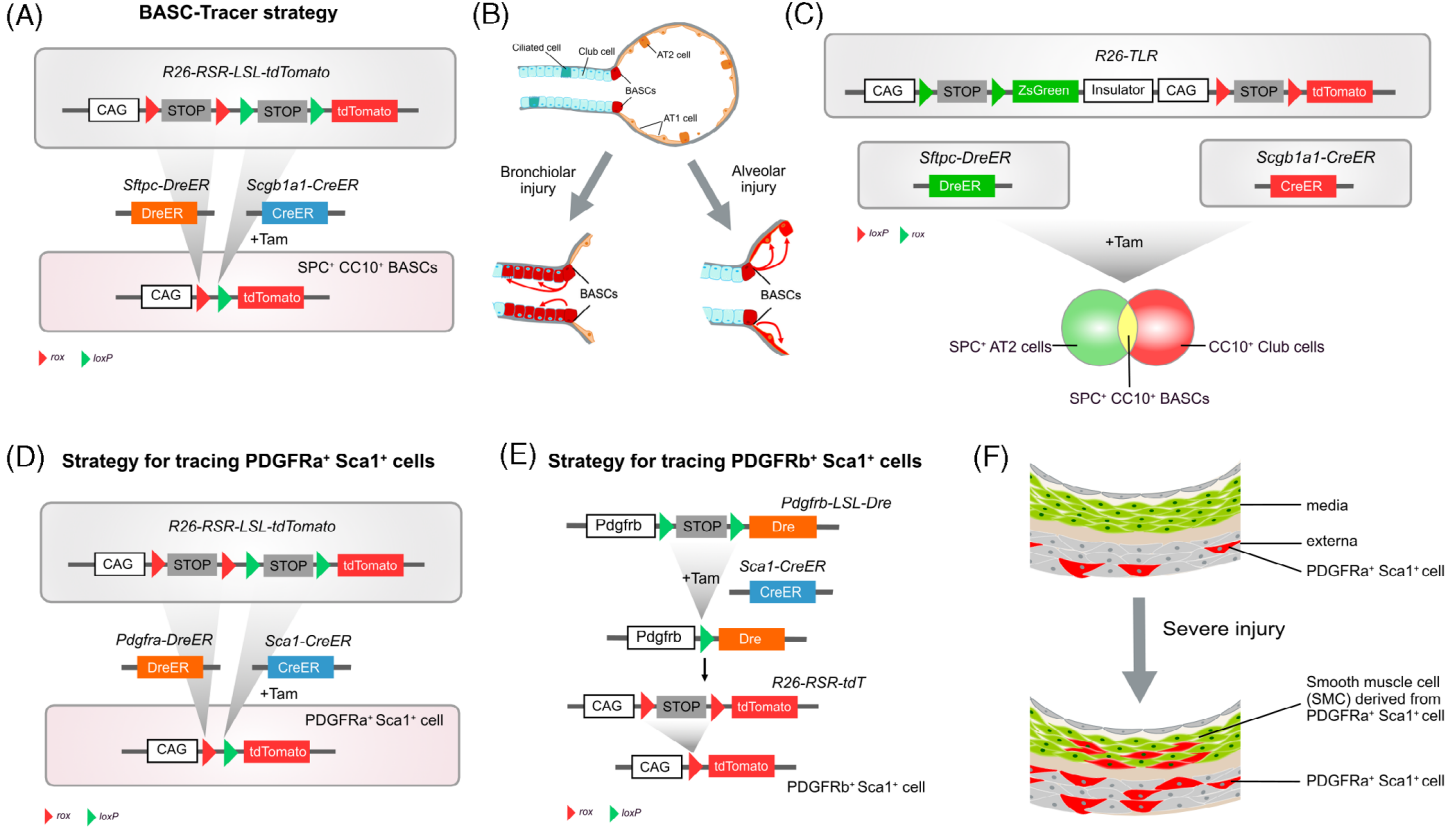
Mechanisms and examples of AND‐logic actualization. (A) The mechanistic illustration shows that only in BASCs where *Sftpc‐DreER* and *Scgb1a1‐CreER* are both active, tdT expression is induced. (B) Bronchioalveolar stem cells contribute to bronchial and alveolar cells in different scenarios. (C) A tandem reporter with ZsGreen and tdTomato induced by Dre and Cre‐mediated recombination, respectively. (D,E) Strategies for tracing PDGFRa^+^ Sca1^+^ cells and PDGFRb^+^ Sca1^+^ cells. (F) PDGFRa^+^ Sca1^+^ cells contribute to vascular SMCs in injury.

To elucidate the role of bronchioalveolar stem cells (BASCs), a potential source of lung regeneration,^34^, ^35^, ^36^ in lung homeostasis and disease, novel genetic tools should be developed to specifically target those functional cells in vivo. Based on Cre and Dre recombinases system, researchers established BASC‐Tracer (*Sftpc‐DreER;Scgb1a1‐CreER;R26‐RSR‐LSL‐tdTomato*) to fate‐map CC10^+^ SPC^+^ BASCs.^37^ Upon Tam induction, only Sftpc (SPC) and Scgb1a1 (CC10) co‐expressed cells could be labelled by tdTomato (Figure 2A). The technology revealed distinct contributions of pluripotent BASCs for lung regeneration in different states, including compensating for club cells and ciliated cells in the bronchioles, and AT1 and AT2 cells in the alveoli (Figure 2B). In addition, club cells, AT2 cells, and BASCs can be distinctly labelled by three fluorescence simultaneously when *Sftpc‐DreER* and *Scgb1a1‐CreER* cross with the reporter line *R26‐TLR*, achieving a synergetic effect greater than the sum of its parts (Figure 2C).^38^ Independently, based on Cre‐*loxP* and Dox‐controlled *TetO*‐tTA system, another study generated BASC v‐race mice, which utilized split‐tTA mediated lineage tracing to label SPC^+^ CCSP^+^ BASCs and their derivatives in several pulmonary disease models.^39^ The study demonstrated the stem cell properties of BASCs in terms of their ability to differentiate across multiple lineages in response to diverse damage.

### 3.2 Generation of de novo smooth muscle cells for artery repair

Rapid vascular repair and regeneration are indispensable for maintaining arterial function after injury. The scRNA‐seq provides plenty of detailed information about the heterogeneity of Sca1^+^ cell sub‐populations and putative Sca1^+^ vascular stem cells.^40^ Based on binary recombinases, intersectional genetics strategies were developed to track two subsets of Sca1^+^ cells. By crossing *Pdgfra‐DreER*, *Sca1‐CreER* with the reporter line *R26‐RSR‐LSL‐tdTomato*, PDGFRa^+^Sca1^+^ could be labelled as tdTomato positive only after both Dre‐*rox* and Cre‐*loxP* recombination upon Tam injection (Figure 2D).^40^ Nevertheless, compared with single‐recombinase‐mediate lineage tracing (*Sca1‐CreER;R26‐tdTomato*), the efficiency of the dual‐recombinase strategy is lower because of the inducible Cre and Dre. Moreover, investigators employed a sequential genetic approach, *Sca1‐CreER;Pdgfrb‐LSL‐Dre;R26‐RSR‐tdTomato*, in which Cre activation leads to Dre‐*rox* recombination, securing tdTomato expression in PDGFRb^+^Sca1^+^ cells (Figure 2E). Fate‐mapping results suggest that PDGFRa^+^Sca1^+^ cells, but not PDGFRb^+^Sca1^+^, in the adventitial layer of artery walls have been shown to differentiate into smooth muscle cells and migrate to the media layer after severe arterial injury (Figure 2F).

## 4 GENETIC PROLIFERATION TRACING BY DUAL RECOMBINASES

Cell proliferation is a typical manifestation of cell fate plasticity. Nucleotide analog incorporation (EdU or BrdU) is a conventional practice in the field. However, accompanying negative side effects including antiproliferation and cell toxicity were reported.^41^, ^42^ Additionally, proliferation markers staining is also extensively used, for instance, cell cycle marker Ki67 (Mki67), cytokinesis marker Aurora kinase B (AuroraB or AURKB), phosphorylated histone H3 (PH3) and proliferating cell nuclear antigen .^43^, ^44^, ^45^ Given that marker staining only reflects snapshots of cell proliferation, rather than continuous traces over a time window, still, signal interference is generated from other proliferating cells. Hence the low signal‐to‐noise ratio of staining methods does not reflect the authentic in vivo cell proliferation situation. While Mosaic Analysis with Double Markers (MADM) system allows record bona fide cell division at the single‐cell level,^46^ the low efficiency of interchromosome recombination underestimates the real proliferation rate.^45^ For lineage tracing, *Ki67‐CreER* was applied to tracing proliferating cells and their descendants; however, considering the inconstant expression of Ki67 in the cell cycle,^47^ it is technically difficult to introduce tamoxifen at the moment of gene activation, thus a subset of proliferating cells could be missed, while persistent tamoxifen administration is toxic to mice. Due to the limitations of current methods, a new genetic tool integrating Dre and Cre recombinases has been designed, ProTracer (Proliferation Tracer), which can non‐invasively and seamlessly monitor in vivo cell proliferation.^48^ Here, we review the adaptation of ProTracer to address academic controversies as well as its strengths and limitations.

### 4.1 Monitoring zonal hepatocyte proliferation in physiology and pathology

**FIGURE 3:**
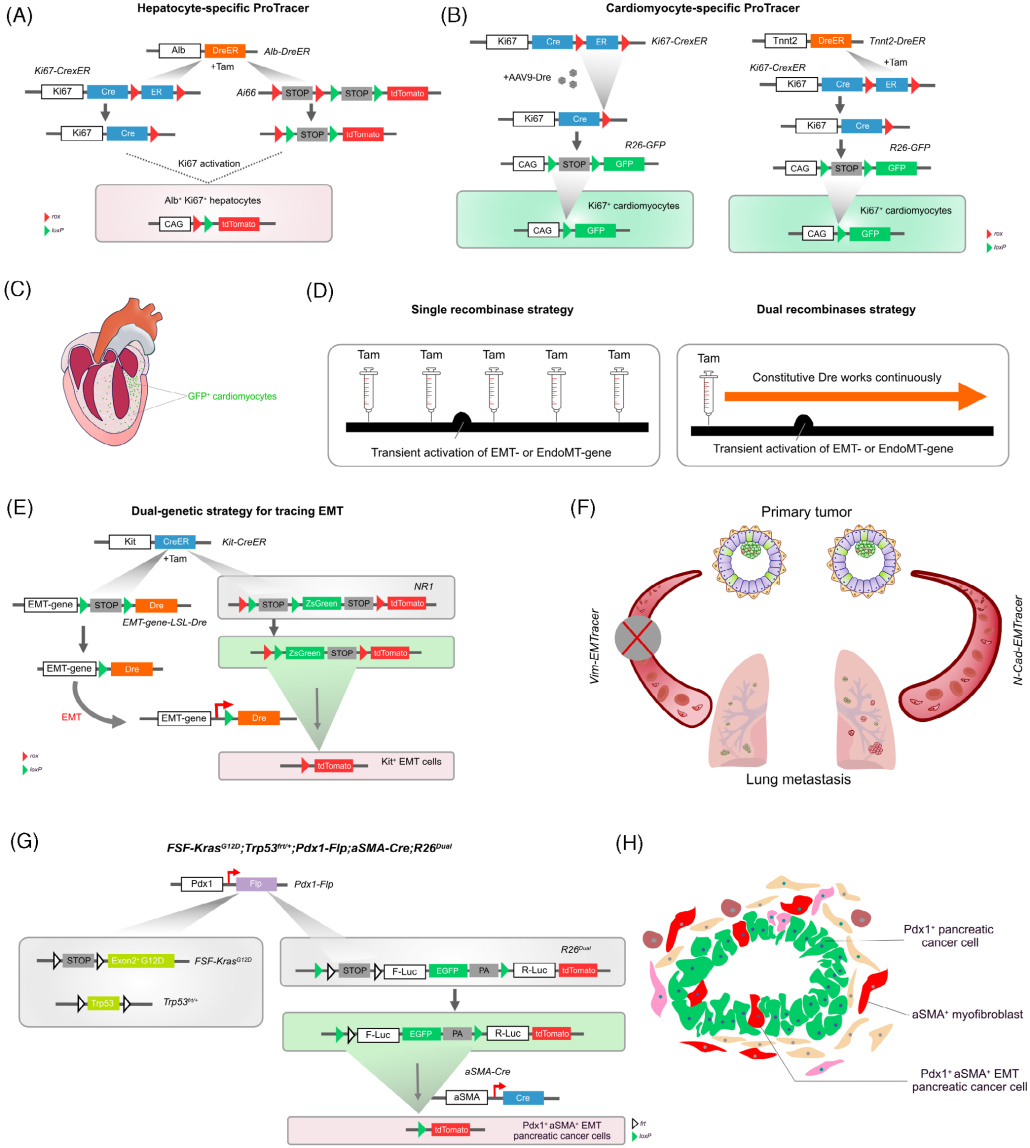
Mechanisms and examples of inducible capturing of transient gene activation. Using (A) *Alb‐DreER* or (B) *AAV9‐Dre/Tnnt2‐DreER*, Ki67 activation can be recorded in hepatocytes or cardiomyocytes upon tamoxifen/AAV administration. (C) Proliferation spatial pattern is shown in the heart. (D) Inducible and continuous recording of gene activation without multiple drug administration. (E) Using *EMT‐gene‐LSL‐Dre* to explore the role of genes in EMT. (F) N‐Cad‐activated, but not Vimentin‐activated breast cancer cells predominantly colonize the lung. (G,H) Cre‐ and Flp‐induced dual‐recombinase system detecting EMT in pancreatic carcinoma.

Self‐renewal of persisting hepatocytes is vital for maintaining the hepatocyte pool.^49^ However, due to the technical limitations of aforementioned methods and the cell heterogeneity in different regions, plenty of conflicting conclusions perplexed the contribution of different hepatocyte populations in liver homeostasis, repair and regeneration.^27^, ^50^, ^51^, ^52^ By incorporating two orthogonal recombinases, the ProTracer technology brings a new interpretation to in vivo hepatocyte proliferation. Theoretically, under a single initial pulse of Tam treatment, hepatocyte‐specific DreER converts the *Ki67*‐*CrexER* to *Ki67‐Cre*. Once Ki67 is activated during proliferation, Ki67^+^ hepatocytes could be permanently labelled by the subsequent expression of the fluorescent reporter (Figure 3A). Combining multiple similar strategies all using *Ki67‐CrexER*, researchers identified midzonal hepatocytes to mainly account for hepatocyte pool maintenance in physiology and the zonal hepatocyte proliferation characteristic in pathology.^48^ In parallel, taking advantage of 11 *CreER* knock‐in mouse models that label diverse zonal subpopulations in liver lobule, and by performing additional in vivo CRISPR knock‐out and activation screening, Wei et al. drew a similar conclusion.^53^ Although the expression of Ki67 represents the entry into the cell cycle, it is not the direct evidence for cell division, as polyploidization and multi‐nucleation events can happen under Ki67 activated without complete cell cycle progression (e.g. in adult mammalian cardiomyocytes).^54^ Thus, to visualize cytokinesis, clonal analysis by sparse labelling has been employed in the ProTracer system.^48^

Compared to the conventional *Ki67‐CreER;R26‐tdT* strategy, the dual‐recombinase‐mediated ProTracer has the following advantages: continuous temporal recording of cell repopulation events, tissue specificity accompanied by a higher resolution ratio, reduced dependence on Tam and thus less cell toxicity, permitting long‐term non‐invasive monitoring of cell proliferation in vivo. It is worth mentioning that in the ProTracer system, not only DreER but also CrexER can respond to Tam induction. However, due to the transient nature of Ki67 expression, the likelihood that both Ki67 expression and Tam induction occur simultaneously in the interested tissue is quite small, and thus the effect is negligible. Besides, activation of the ProTracer system is time‐consuming and, as detailed in the study, it takes several weeks to achieve a stable detection state in liver homeostasis. Moreover, it is inexplicable to figure out regional repopulation features in highly proliferative tissues. Conversely, ProTracer is especially adaptive in rarely proliferative cell lineages, for example, cardiomyocytes.^48^ Thus, to illustrate the utility of the technique, we will discuss the application of ProTracer to cardiomyocyte proliferation in different phases in the following subsections.

### 4.2 Tracking cell proliferation at multiple stages in the heart

Nowadays, a broad consensus has been reached that new cardiomyocytes are generated from self‐proliferation.^55^ To investigate cardiac regeneration in adult heart, the cardiomyocyte‐tropistic AAV9‐Dre primed ProTracer system was constructed, in which Dre‐*rox* recombination switches cell‐cycle marker *Ki67‐* or *Ccna2‐*driven *CrexER* to *Ki67‐Cre* or *Ccna2‐Cre*, respectively, thereafter, permanently labels Ki67^+^ or Ccna2^+^ cells by the GFP reporter.^56^ To avoid region‐preferential viral infection, *Tnnt2‐DreER*‐triggered cardiomyocyte‐specific ProTracer was also generated (Figure 3B). By exploiting multiple strategies, the regional distribution pattern of cycling cardiomyocytes in homeostasis and injury was revealed in the adult heart (Figure 3C). Moreover, ProTracer has been adapted to reveal cardiomyocyte proliferation patterns in the developing mammalian heart. Utilizing CAG‐Dre primed ProTracer (*R26‐DreER;Ki67‐CrexER;Tnnt2‐mTnG*) to seamlessly label Ki67^+^ cardiomyocyte cells, a rapid cell‐cycle withdrawal in cardiomyocytes from birth to adolescence was proved,^57^ disproving the previous conclusion that cell burst proliferation occurs during preadolescence.^58^

Although the ProTracer system permits cycling cardiomyocyte labelling, it is unable to illustrate the progenitors of those proliferative cardiomyocytes. Additionally, as noted above, Ki67 or Ccna2 marked cell proliferation scarcely distinguishes polyploidization and cytokinesis events. However, the MADM system visualizes cell division by labelling two daughter cells as red and green separately, even though the efficiency of interchromosome recombination needs to be improved.^46^ Possibly in combination with the MADM system, the ProTracer system could be advanced in the future to record cell proliferation more precisely by continuously capturing authentic in vivo cell division events.

## 5 SEAMLESSLY RECORDING GENE ACTIVATION DURING CELL TRANS‐DIFFERENTIATION

In vivo gene expression patterns are complex and changeable in diverse biological processes.^59^ As for recording the expression of a particular gene, a single inducible recombinase system is unable to constantly operate without drug induction, and it is difficult to identify the gene over a time window through a constitutive single‐recombinase strategy. Meanwhile, tissue specificity is hard to accommodate. However, the proposed dual‐recombinase system circumvents the problems above. Similar in theory to ProTracer, it converts an inducible recombinase to a constitutive one, hence permitting continuous recording of gene activation in specific cell types, especially for the short‐term or transiently expressing genes (Figure 3D). Not only for proliferation as described above, but here we also discuss the application of the dual‐recombinase system to seamlessly capture gene activation during cell transdifferentiation.

### 5.1 Detection of capillary to coronary artery formation in the adult heart

As a marker of angiogenesis, Apelin (APLN) is highly expressed in sprouting capillaries but it is challenging to label APLN^+^ capillaries in the adult heart as its decreased expression.^60^ Besides, the indistinguishable labels of all capillaries have low signal‐to‐noise ratios. Turning to the dual‐recombinase system, under the initial Tam treatment, the inducible *Apln‐CrexER* is converted to the constitutive *Apln‐Cre* by *Cdh5‐DreER*, thus incessantly records the activation of *Apln* in CDH5^+^ capillary endothelial cells (ECs). Then, the GFP could be activated if APLN^+^ ECs contribute to artery formation driven by *Cx40* expression. By employing this strategy, investigators demonstrated that capillary ECs contribute to arteries in the adult heart after myocardial infarction.^61^

### 5.2 Capturing EMT activity during tumour metastasis

It is widely acknowledged that epithelial‐to‐mesenchymal transition (EMT) occurs in diverse processes including embryonic development and pathological conditions such as tumorigenesis, invasion and metastasis.^62^, ^63^ Given that EMT is transient and reversible,^64^ the conventional single‐recombinase lineage tracing strategy may not accurately record the EMT programme. That means using EMT‐gene‐driven *CreER* with a reporter line or by immunofluorescent staining for mesenchymal markers in lineage‐tagged cancer cells.^65^ To precisely visualize in vivo EMT in the metastatic cascade, researchers developed a novel genetic approach based on Cre and Dre dual recombinases to seamlessly record EMT gene activation. In the system, *MMTV‐PyMT* induces breast adenocarcinoma tumorigenesis spontaneously.^66^ After Tam induction, Kit^+^ mammary luminal epithelial cells are labelled as ZsGreen after Cre‐*loxP* recombination with the *nested reporter* (*NR1*). Meanwhile, *Kit‐CreER* removes the *loxP*‐flanked *stop* cassette before *Dre*, then a constitutive *Dre* can seamlessly monitor EMT gene activity in Kit^+^ luminal epithelial cells. Once EMT gene *Vimentin* or *N‐cadherin* is activated in tumour metastasis, Dre‐*rox* recombination blocks ZsGreen expression, and irreversibly converts the label from ZsGreen to tdTomato (Figure 3E). The EMTracer model is temporal‐controlled, tissue‐specific and with a strong resolution, proving that *N‐cadherin* is functionally required during breast‐to‐lung tumour metastasis, instead of *Vimentin* (Figure 3F).^67^, ^68^

Cre‐ and Flp‐coupled dual‐recombinase systems were also established to detect EMT in pancreatic carcinoma.^69^, ^70^ *FSF‐Kras* ^*G12D/+*^ *;Trp53* ^*frt/+*^ *;Pdx1‐Flp* (*KPF*) mouse model was generated to induce pancreatic ductal adenocarcinoma (PDAC). When the KPF mouse line is crossed with *aSMA‐Cre* and *R26* ^*Dua*l^, Pdx1^+^ cancer cells would be labelled as EGFP^+^ by Flp‐*frt* recombination, upon acquisition of mesenchymal features including aSMA activation, those cells would permanently express tdTomato even if subsequent mesenchymal‐to‐epithelial transition (MET) process (Figure 3G).^69^ However, in *KPF;aSMA‐Cre;R26* ^*Dual*^ mice, either aSMA^+^ myofibroblasts or aSMA^+^ EMT cancer cells could be marked as tdTomato (Figure 3H), thus requiring epithelial cancer cell markers to distinguish them. In addition, it is difficult to explain whether EMT in embryonic development may interfere with the results due to constitutive Cre being driven by aSMA. However, another mouse model of PDAC, *FSF‐Kras* ^*G12D/+*^ *;Pdx1‐Flp;FSF‐R26* ^*CAG‐CreERT2*^, is capable of temporal control. As the readout of Flp‐*frt* recombination is CreER, the inducible Cre can be used for genetic manipulation in the *Pdx1‐Flp* lineage, including gene knockout and cell depletion in a time window.^70^

### 5.3 Recording mesenchymal gene activation in cardiac fibrosis

To testify whether myofibroblasts are derived from endothelial to mesenchymal transition (EndoMT) in adult cardiac fibrosis, investigators crossed *Cdh5‐CreER*, *aSMA‐LSL‐Dre* and aforementioned *NR1*, generating aSMA‐EndoMTracer.^71^ After Tam induction, Cre‐*loxP* recombination yields ZsGreen^+^ ECs and the *aSMA‐Dre* allele. Transient aSMA activation enables the switch from ZsGreen to tdTomato via Dre‐*rox* recombination, and the ECs could be irrevocably labelled as tdTomato^+^ even if they subsequently transdifferentiate into mesenchymal cells. Together with the Zeb1‐EndoMTracer, EndoMT was illuminated in embryonic heart valve formation, but not in adult cardiac fibrosis. Parallelly, *Col1a2‐CreER;aSMA‐LSL‐Dre;NR1* demonstrated that fibroblasts contribute to cardiac fibrosis.^71^ However, a caveat of the above approach is the crosstalk of *Cdh5‐CreER* with *NR1*, where Cre targets *rox* sites and unlocks the expression of tdTomato independent of aSMA activation. In order to circumvent the potential risks, strict control should be established under the same experimental conditions.

## 6 IN VIVO GENE MANIPULATION BY THE DUAL‐RECOMBINASE SYSTEMS

Tissue‐specific conditional mutants have been extensively used to study gene function in vivo during development, homeostasis or pathological states. Typically, the vital element (e.g. exons) of the target gene is flanked by two unidirectional *loxP*, and the gene becomes non‐functional after Cre site‐specific recombination.^4^ Compared with germline null mutations, tissue‐specific gene deletion can circumvent embryo lethality occasionally; moreover, temporal control of conditional mutagenesis (e.g. by CreER) identifies gene functions at a particular time point.^72^ However, neither the inducible nor the constitutive single‐recombinase system is perfect for gene deletion, as the former is temporal but inefficient, and the latter is efficient but non‐temporal. And both of them are limited by a single promoter. It is better to combine their merits and overcome their defects at the same time. Hence, a gene‐manipulating dual‐recombinase system was developed for high‐resolution conditional mutagenesis.

### 6.1 Dual‐genetic strategies for gene knockout

Orthogonal dual‐SSR strategies, such as Cre and Dre, Cre and Flp, have been engineered to analyse gene functions. As the leakage of *rox‐stop‐rox‐Cre* in the absence of Dre, Roxed‐Cre was constructed, in which the *Cre* ORF was interrupted by a roxed‐long *stop* cassette between amino acids 59 and 60.^73^ By electroporation, plasmids encoding *hGFAP‐Dre*, *Thy1.2‐Roxed‐Cre (or Thy1.2‐Dre*, *hGFAP‐Roxed‐Cre)* and constitutive EGFP were delivered to the brain of E14.5 embryo, and the embryo also carrying a Cre‐inducible tdTomato reporter gene.^73^ The Cre recombination outputs resulted in fluorescent labelling of distinct cell populations in the cortex of mice. The study has shown that the dual‐SSR system has the potential of harbouring tissue specificity and temporal layers of control, thus significantly increasing the resolution of lineage tracing. However, plasmid transfection efficiency may cause the results open to bias, and the efficiency of reporter gene expression does not always faithfully mirror the efficiency of gene knockout.^72^ As for improving the efficiency of gene deletion, more split sites between CreN and CreC should be tried, and more solid in vivo genetic evidence from the roxed‐Cre knock‐in mice is needed. The dual‐recombinase‐mediated gene manipulation technology should be optimized in terms of timing regulation, specificity and efficiency.

**FIGURE 4:**
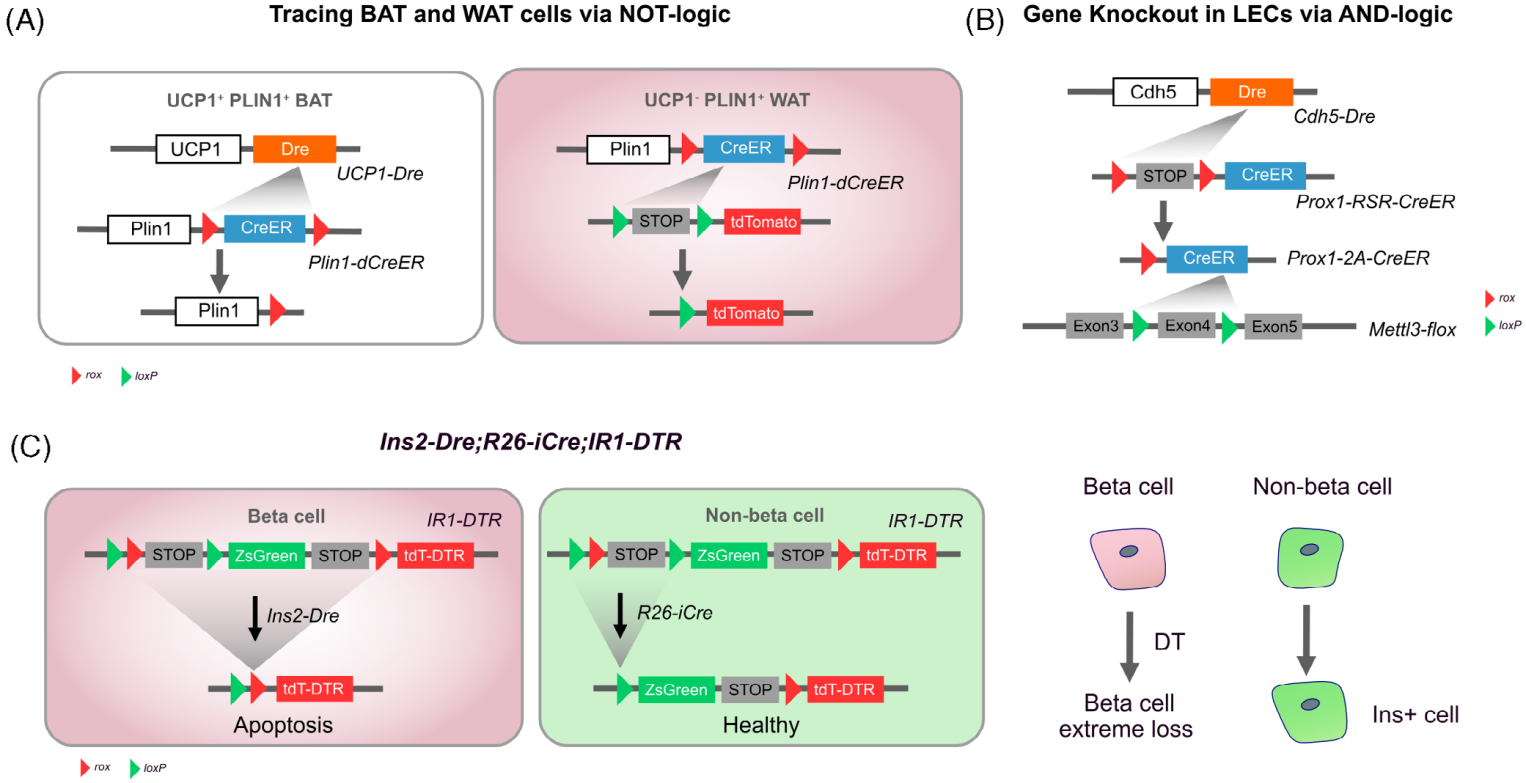
Mechanisms and examples of genetic manipulation with dual recombinases. (A) To activate Cre in WAT but not BAT, *Plin1‐dCreER* is designed to allow *UCP1‐Dre* to remove the *CreER* sequence in BAT. (B) *Prox1‐RSR‐CreER* is designed to be activated by endothelial‐specific *Cdh5‐Dre*. (C) With *IR1‐DTR*, beta cells can be ablated after DT administration, and ZsGreen^+^ non‐beta cells can transdifferentiate into insulin‐expressing cells.

A novel dual‐recombinase system involving Dre and inducible Cre has been developed to perform specific and efficient gene deletion in vivo in white adipose tissue (WAT), in which pan‐adipocyte marker PLIN1 and brown adipocyte tissue‐ (BAT) specific marker UCP1 was used.^74^ In the presence of Dre driven by *Ucp1*, the *rox‐*flanked *CreER* was removed in *Plin1‐rox‐CreER‐rox* (*Plin1‐dCreER*) mice (Figure 4A). Since Cre was the readout in WAT in this intersectional genetic system, *Plin1‐dCreER;Ucp1‐Dre;Pparg* ^*fl/fl*^ mice were generated to knock out the gene *Pparg* in WAT specifically but not BAT.^74^ Compared with traditional pan‐adipocyte gene ablation,^75^ this system enables more specific and efficient gene deletion. In addition to the NOT‐logic, AND‐logic strategy, *Cdh5‐Dre;Prox1‐RSR‐CreER;Mettl3* ^*fl/fl*^ mice were also generated to specifically delete the gene *Mettl3* in CDH5^+^ PROX1^+^ lymphatic ECs (LECs) (Figure 4B). As *Prox1* is also expressed in other cell lineages,^76^, ^77^ using the conventional *Prox1‐CreER* line to target LECs may confound the interpretation of gene functions, whereas the dual‐genetic strategy improves the accuracy of gene knockout by enhanced tissue specificity. Taken together, more than acting as a powerful lineage‐tracing tool, the dual‐recombinase systems also perform high‐resolution genetic manipulation that is unattainable with the single‐recombinase systems.

### 6.2 Cellular function studies via the dual‐recombinase systems

In order to obtain functional information at the cellular level, typically genes associated with cell death, pyroptosis, or apoptosis are endogenously expressed. For instance, GSDMD N‐terminal (GSDMD‐NT) could induce pyroptosis,^78^, ^79^ and P21 induces cell proliferation inhibition and senescence.^80^, ^81^ Besides, chemical‐genetic cell ablation strategies such as the NTR‐MTZ system in zebrafish, and the DT‐DTR system in mammals also work efficiently under spatial and temporal control.^82^, ^83^

Since the non‐specific labelling by a single marker may lead to misunderstanding,^84^, ^85^, ^86^ researchers took advantage of *Ins2‐Dre;R26‐iCre;IR1‐DTR* mice to distinguish beta cell and non‐beta cell by labelling them as tdT‐DTR^+^ and ZsGreen^+^, respectively (Figure 4C). After DT administration, the vast majority of beta cells (>99%) were genetically cleared. The *IR‐DTR* system captured a few ZsGreen^+^ cells contributing to a subset of insulin^+^ cells, meanwhile, indicating the sensitivity of the system.^87^ Together, the combination of DT‐DTR and the intersectional genetic manipulation approach further demonstrates the broad compatibility of the dual‐recombinase system.

## 7 CONCLUSION AND DISCUSSION

As mentioned above, the dual‐recombinase system exhibits its potent faculty due to the improved spatiotemporal specificity, higher efficiency and resolution in numerous scientific issues that conventional single‐recombinase systems cannot achieve. Not only does it avoid the risk of unnecessary labelling by single promoter‐driven recombination, but also it is able to continuously monitor transient gene activation during cell proliferation and cell fate transitions. Furthermore, more specific in vivo genetic manipulation can be achieved due to the accurate targeting of different cell populations by the dual‐recombinase approaches.

As a system depending on transgenic animals and SSRs, the dual‐recombinase system inherits the challenges of SSRs in the way of faithfully reflecting endogenous molecular expression map, and probably exacerbates the issues due to its increased complexity. The recombination efficiency, tissue specificity,^88^ risk of spontaneous activation,^89^ as well as the disparity and cross‐activation among different SSRs in dual‐recombinase systems^90^ could all lead to false conclusions, although dual‐recombinase systems do extend the capability of genetic manipulations using SSRs when they are well‐tuned. Unpredictability exists in all steps from genetic design to individual animal variations, making it extremely delicate to get satisfactory results. Consequently, recombinase should be designed and induced to activate at a balanced level to prevent either leaky or poor recombination efficiency, each mouse line should be well characterized with sufficient biological replicates and appropriate control groups should be set up in the experimental design.

Besides its limitations to be overcome, what might be the future of dual‐recombinase systems? We have seen the development of single‐cell lineage tracing based on different genetic editing tools, including Polylox barcodes edited by Cre recombinase,^91^ CRISPR‐Cas9, TdT,^92^ prime‐editing guide RNA, etc.,^93^ which have much higher throughput at the single‐cell level and allow data‐driven rather than hypothesis‐driven research compared to conventional lineage tracing.^94^ However, these technologies require multiple large genetic modules, are not well‐controlled in vivo, contain a certain amount of noise information, or have a low editing efficiency that impedes a clear interpretation of the data.^95^ It could be very rewarding if we combine the dual‐recombinase system with single‐cell lineage tracing to control the expression of these above‐mentioned machinery in vivo with both tissue and temporal resolution remained.

With the development of omic technologies like deep sequencing, spatial transcriptomics and mass spectrometry, genetic systems with SSRs can be enhanced with dual‐recombinase systems. New marker genes of specific cell subtypes locked on by dual recombinases can be identified by deep sequencing. Proximal cell and molecule profiling by gLCCC/gTCCC^96^ and proximity labelling^97^ that utilizes synNotch and HRP modules can be tamed by dual recombinases and combined with spatial transcriptomics or proteomics to unveil in vivo and in situ cell–cell interaction and the underlying molecular mechanisms. Moreover, live imaging with cell fate mapping allows for real‐time and in situ inspection of cellular processes and can facilitate the use of photoactivatable recombinases for light‐controlled recombination with tissue and temporal constraints.^98^, ^99^ Besides, the application of the dual‐recombinase system in the fields of cell origin, proliferation and differentiation has provided conclusive evidence for cell fate plasticity until now, yet has barely penetrated the fields of cell senescence, apoptosis or cell death. Therefore, further iterations of this genetic approach are urgently needed to broaden its reach to interpret the entire life of cells and address more unsettled scientific questions in biological processes.

By integrating different SSRs, dual‐recombinase‐mediated lineage tracing and manipulation provide higher spatiotemporal resolution than single recombination strategies, while also having strong compatibility to cooperate with other technologies to expand its applications.

## AUTHOR CONTRIBUTIONS

Hongxin Li drafted the paper and schematics, and Wendong Weng contributed the cartoons in figures and provided intellectual input. Bin Zhou revised the article.

## FUNDING INFORMATION

This work was supported by the Strategic Priority Research Program of the Chinese Academy of Sciences (CAS grant XDA16010507); National Key Research & Development Program of China (2019YFA0110404, 2019YFA0802000).

## CONFLICT OF INTEREST STATEMENT

No conflict of interest.

## Data Availability

Data sharing is not applicable to this article as no new data were created or analyzed in this study.
